# Antioxidant and Anticancer Activities of Water Extracts from Flowers, Leaves and Stems of In Vitro Cultivated and Wild-Growing *Marrubium vulgare* Plants

**DOI:** 10.3390/ph18121806

**Published:** 2025-11-26

**Authors:** Krasimira Tasheva, Ani Georgieva, Inna Sulikovska, Maria Petrova, Margarita Dimitrova, Lyudmila Dimitrova, Elena Georgieva, Petko Denev, Maria Lazarova, Polina Petkova-Kirova

**Affiliations:** 1 Institute of Plant Physiology and Genetics, Bulgarian Academy of Sciences, Acad. G. Bonchev Str., 21, 1113 Sofia, Bulgaria; marry_petrova@yahoo.com (M.P.); mstoyadinova@abv.bg (M.D.); dim.lyudmila@gmail.com (L.D.); elenaiv359@abv.bg (E.G.); 2 Institute of Experimental Morphology, Pathology and Anthropology with Museum, Bulgarian Academy of Sciences Acad. G. Bonchev Str., 25, 1113 Sofia, Bulgaria; georgieva_any@abv.bg (A.G.); inna_sulikovska@ukr.net (I.S.); 3 Institute of Organic Chemistry with Centre of Phytochemistry, Bulgarian Academy of Sciences, Laboratory of Biologically Active Substances, 4000 Plovdiv, Bulgaria; petko.denev@orgchm.bas.bg; 4 Institute of Neurobiology, Bulgarian Academy of Sciences, Acad. G. Bonchev Str., 23, 1113 Sofia, Bulgaria; m.lazarova@gmail.com

**Keywords:** *M. vulgare*, micropropagation, anticancer activity, apoptosis, cell cycle arrest

## Abstract

**Background/Objectives:** *Marrubium vulgare* L. is a medicinal plant with diverse pharmacological properties, yet its in vitro cultivation and the biological potential of aqueous extracts of the plant remain poorly studied. The present research aimed to establish an efficient in vitro propagation protocol and to compare the antioxidant and anticancer activities of freeze-dried water extracts from different parts (leaves, flowers, and stems) of in vitro cultivated and wild-growing *M. vulgare* plants. **Methods:** A micropropagation system was developed using Murashige and Skoog medium supplemented with kinetin and indole-3-acetic acid. Extracts from leaves, flowers, and stems were evaluated for the total polyphenol and flavonoid content, antioxidant capacity (ORAC, HORAC), and antiproliferative effects against HeLa, HT-29, and MCF-7 cancer cell lines. The mechanism of cytotoxicity was examined through apoptosis and cell cycle analysis. **Results:** The established protocol achieved high propagation efficiency (90% shoot formation). Cultivated leaves showed the highest polyphenol and flavonoid content and the strongest antioxidant activity. Aqueous extracts, particularly from leaves and flowers, displayed selective antiproliferative effects with HeLa cells being the most sensitive. The extracts induced apoptosis and cell cycle arrest –mainly at the G1 phase for cultivated plants and at both G1 and G2/M phases for wild plants. **Conclusions:** An efficient micropropagation protocol was successfully developed, providing a sustainable source of biologically active plant material. The study provides the first comprehensive comparison of *M. vulgare* water extracts from in vitro cultivated and wild-growing plants, linking phytochemical content with antioxidant and anticancer properties and highlighting both wild and in vitro cultivated plants, though wild plants in certain cases are generally more efficient, as promising candidates in natural anticancer therapeutics. The elevated flavonoid levels in in vitro cultivated plants, together with enhanced antioxidant capacity, indicate the strong potential of in vitro cultivated plants in antioxidant and cytoprotective formulations for cardiovascular, neurodegenerative, and metabolic diseases.

## 1. Introduction

Species from the Lamiaceae family are widely used for the prevention and treatment of a number of diseases, as well as in the pharmaceutical and perfume industries. The plant *Marrubium vulgare* L., commonly known as white horehound, is a perennial herb found throughout Southwest and Central Asia, North Africa, and Europe, including Bulgaria, especially in grassy and stony areas [1]. The plant is widely used in traditional (folk) medicine for lung, respiratory, digestive, and other infections. Extensive phytochemical studies on *M. vulgare* have identified a diverse array of 54 secondary metabolites such as diterpenes, sesquiterpenes, flavonoids, phenylpropanoids, and others, in different parts of the plant, with antioxidant, anti-inflammatory, and antimicrobial properties underlying its hepatoprotective, antidiabetic, antihypertensive, and cardioprotective effects [2]. Marrubiin, marrubinic acid, and marrubenol are major diterpenes that exhibit analgesic and anti-edematogenic activity. In addition, studies have highlighted the anticancer activity of *M. vulgare* as well. Extracts of *M. vulgare* and bioactive compounds isolated from the plant such as flavonoids like the flavons apigenin, acacetin, and ladenein have demonstrated cytotoxic effects against various cancer cell lines, suggesting that *M. vulgare* may serve as a promising candidate for further oncological research and drug development. Thus, Nawal and Atta [3] have demonstrated the anticancer effect of *M. vulgare* ethanol extracts as well as that of six pure flavonoid compounds from the plant on Ehrlich tumor cell lines (U251) and breast cancer cell lines (MCF-7). Moreover, Belayachi et al. [4] have shown the antiproliferative activity of the methanol concentrated extract of the plant, next dissolved in water and successively extracted with dichloromethane, against human glioblastoma (T-98Gand U87GM), colorectal cancer (SW620), prostate cancer (PC-3 cells), and with especially high efficiency against acute T-cell leukemia (Jurkat cells) and Mantle cell lymphoma (Jeko-1 cells). In their report, Yamaguchi et al. [5] further confirmed the antiproliferative activity of the extracts of *M. vulgare* leaves in human colorectal cancer cells through the suppression of cell growth as well as the induction of apoptosis via the up-regulation of NAG-1 (the pro-apoptotic non-steroidal anti-inflammatory drug-activated gene) through the transactivation of the NAG-1 promoter. The methoxylated flavone ladanein, isolated from *M. vulgare*, was also shown to display antiproliferative activity against human leukemia cells (K652, K562R) [6]. Cervical cancer is also among the cancers sensitive to *M. vulgare* as its essential oil was shown to inhibit the proliferation of HeLa cells [7].

The technique of in vitro micropropagation has established itself as a powerful tool in plant tissue cultures for the mass production of large quantities of plants in a short period of time [8] for the purpose of preserving wild species and improving the synthesis of secondary metabolites [9,10]. Despite the great interest in *M. vulgare* and extensive research on its phytochemical composition, elaborate in vitro methods for its propagation are very limited. In the literature, there are just a few reports on the in vitro cultivation of the species [11,12,13]. Therefore, further research on the in vitro propagation of *M. vulgare* is essential to refine cultivation protocols and optimize nutrient media and plant growth regulators’ additives to develop reliable regeneration systems. Furthermore, several studies have shown significant variations in the secondary metabolite content of *M. vulgare* in dependence of origin, environmental conditions, and different growth stages [14,15], which emphasizes the necessity for plant material with a consistent constant quality of active components, achievable through in vitro propagation.

Thus, the aim of the present study was to develop an efficient protocol for the in vitro propagation of *M. vulgare* and to evaluate and compare the antioxidant and anti-tumor activities of different parts—leaves, flowers, and stems—of the in vitro obtained and cultivated plants and their wild-growing counterparts.

## 2. Results

### 2.1. In Vitro Culture

#### 2.1.1. Shoot Multiplication

The seed surface sterilization procedure effectively produced fully decontaminated seeds, successfully establishing an aseptic culture. High germination rates were observed on Murashige and Skoog (MS) [16] basal medium without the addition of plant growth regulators (PGRs), reaching 65% by the 21st day. Nodal segments obtained from one-month-old in vitro-grown seedlings exhibited strong regenerative capacity, making them well-suited for initiating the micropropagation of *M. vulgare*.

**Figure 1:**
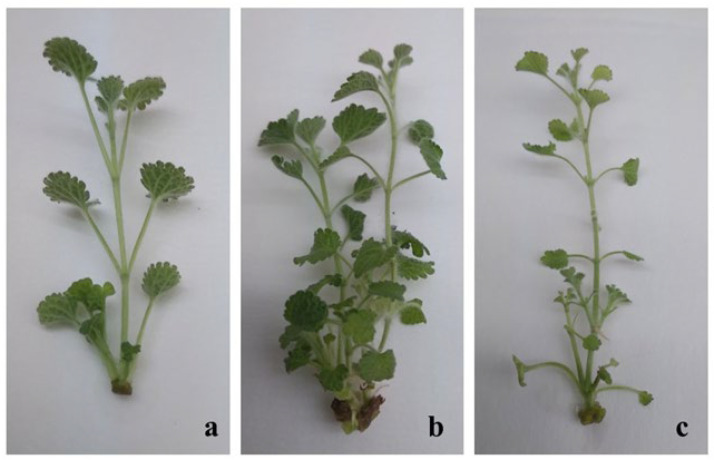
Micropropagation of *M. vulgare* L. on nutrient media supplemented with different plant growth regulators: (**a**) MS supplemented with 1 mg/L zeatin and 0.1 mg/L IAA, (**b**) MS supplemented with 1 mg/L kinetin and 0.1 mg/L IAA, (**c**) MS supplemented with 1 mg/L BAP and 0.1 mg/L IAA (in vitro micropropagated plants were photographed outside the cultivation vessels).

The control MS medium free of plant growth regulators did not yield new shoots during the tested culture period. The inoculated shoots only increased in height. Initial explants showed varying micropropagation potential based on the type and concentration of the plant growth regulators used. When testing the effectiveness for micropropagation of *M. vulgare*, of the cytokinin kinetin added at various concentrations to the culture medium, it was observed that the number of newly formed shoots varied between 2.75 and 3.08. The highest multiplication frequency (95%) was achieved on MS medium containing the highest kinetin amount (2 mg/L). However, shoots raised on nutrient medium with 1 mg/L of kinetin reached the biggest elongation compared to those cultured on media containing higher kinetin amounts. Nutrient media supplied with both cytokinins and auxins were further used to promote multiplication efficiency. The media contained one cytokinin, zeatin, 6-benzylaminopurine (BAP), or kinetin, and in some cases, was combined with one of three auxins: indole-3-butyric acid (IBA), α-naphthalene acetic acid (NAA), or indole-3-acetic acid (IAA). The best results were obtained on MS nutrient medium supplemented with kinetin (1.0 mg/L) and IAA (0.1 mg/L), where 90% of the explants formed new shoots, with an average number of shoots per explant of 4.0 and an average height of 3.70 cm after 4 weeks of culture (Table 1; Figure 1b). Shoots can be divided from shoot clumps, with each shoot containing 3–4 nodes. This procedure allows for the generation of multiple explants for subsequent experiments. Nutrient media in which kinetin was combined with other types of auxins exhibited decreased efficacy. The media containing zeatin Z_1_I_0.1_ (Figure 1a) and 6-benzylaminopurine B_1_I_0.1_ (Figure 1c) were less effective for the micropropagation of this plant species. The measured fresh and dry weight (DW) of the samples was as follows: on medium K_1_I_0.1_—0.170 g:0.055 g; K_2_—0.184 g:0.043 g; K_1_—0.034 g:0.014 g.

#### 2.1.2. In Vitro Rooting, Adaptation, and Acclimatization Ex Vitro

**Figure 2:**
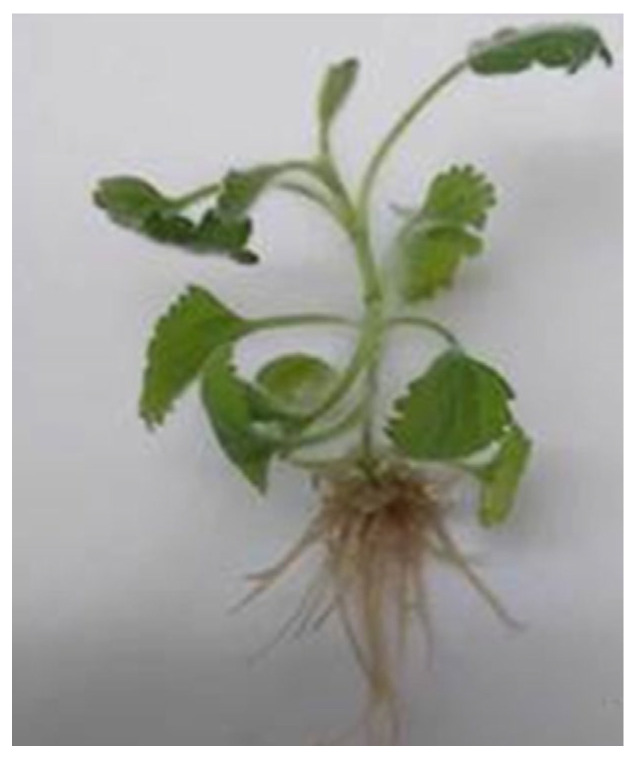
In vitro rooting of *M. vulgare* on half strength MS medium augmented with 2 mg/L IBA and 0.2 mg/L IAA.

Root initiation was observed in 5–6 days of culture on all tested media. The medium supplemented with 2 mg/L IBA and 0.2 mg/L IAA resulted in the highest rooting frequency (90%) and the maximum number of roots (6.4) (Table 2, Figure 2). The in vitro shoots grown on this medium achieved an average root length of 1.3 cm after 4 weeks of culture. The second effective medium was MS medium containing 1 mg/L IBA and it ensured 70% root induction. The nutrient medium supplemented with 0.1 mg/L IBA was the least effective.

**Figure 3:**
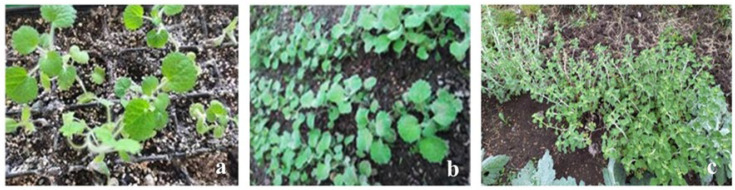
Adapted plants *M. vulgare*: (**a**,**b**) plants acclimatized in pots; (**c**) plants adapted in the field.

The gradual acclimatization of the in vitro-derived plants to external conditions facilitated their successful adaptation. The substrate used for this process consisted of peat, perlite, sand, and soil mixed in a 2:1:1:1 ratio, supporting a survival rate of up to 70%. The hardening process was carried out in a greenhouse over the course of one month. No morphological differences were detected in the plants following ex vitro adaptation (Figure 3). The adapted plants were successfully established in the “Gorna Baniya” fields (800 m altitude) in the region of Sofia with the majority (80%) of plants flowering during the first year.

### 2.2. Content of Total Polyphenols, Total Flavonoids, and Antioxidant Activity

The highest content of total polyphenols and flavonoids, as well as antioxidant activity using the oxygen radical absorbance capacity (ORAC) and hydroxyl radical averting capacity (HORAC) methods, was measured in the leaves of cultivated plants, followed by leaves from wild species (Table 3).

The highest marrubiin content was observed in the flowers of wild plants, followed by that in the flowers of cultivated plants, whereas the lowest content was detected in the stems of cultivated plants (Table 4).

### 2.3. Assessment of Anticancer Activity

#### 2.3.1. Antiproliferative Activity

**Figure 4:**
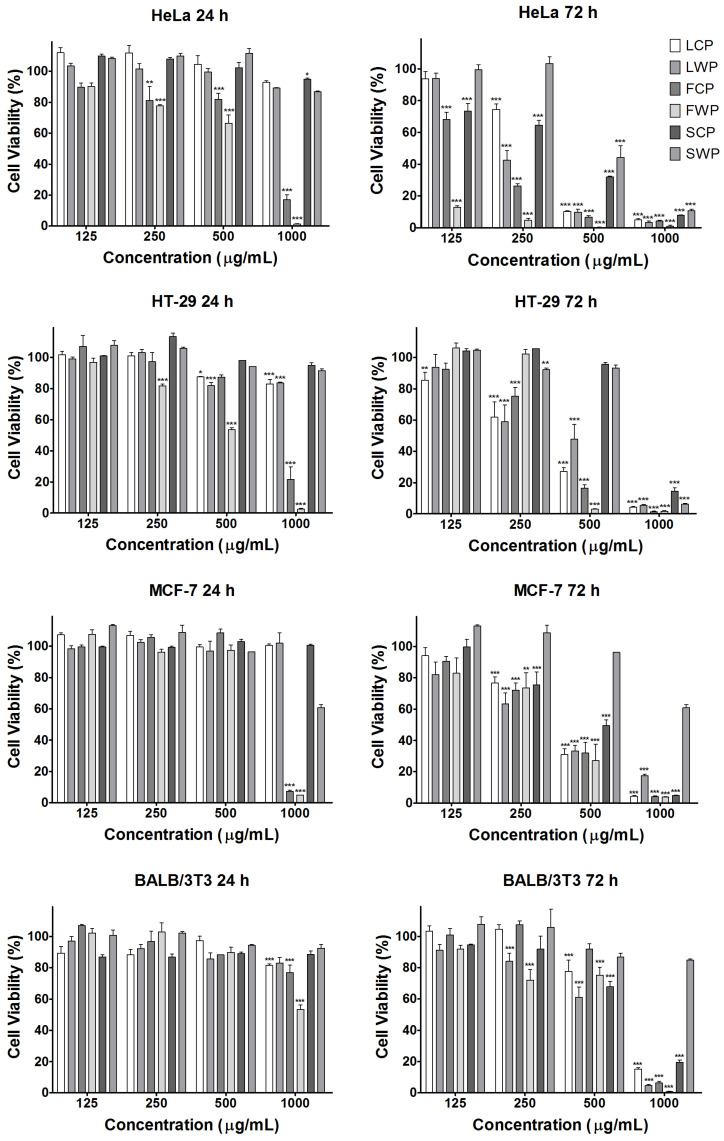
Antiproliferative effects of *M. vulgare* extracts assessed by the MTT test after 24 and 72 h of exposure. FCP (flowers of in vitro cultivated plants); FWP (flowers of wild plants); LCP (leaves of in vitro cultivated plants); LWP (leaves of wild plants); SCP (stems of in vitro cultivated plants); SWP (stems of wild plants). Values are presented as means ± SD; * *p* < 0.05, ** *p* < 0.01, and *** *p* < 0.001.

The antiproliferative effect of the different *M. vulgare* extracts was evaluated in the cancer cell lines HeLa, HT-29, and MCF-7 and the non-tumorigenic cell line BALB/3T3 after 24 and 72 h using the 3-(4,5-dimethylthiazol-2-yl)-2,5-diphenyltetrazolium bromide assay (MTT) test for cell viability and proliferation (Figure 4).

Based on the results of the MTT analysis, the IC_50_ concentrations of the extracts for each of the tested cell lines were determined and are presented in Table 5.

Comparing the effects of the extracts obtained from the different anatomical parts of *M. vulgare* plants, leaf and flower extracts showed significantly higher activity compared to stem extracts in all cell lines tested. The antiproliferative activity of the flower extracts was much higher than that of the leaf extracts in the HeLa cell line at both studied time intervals and in HT-29 cells treated for 24 h. After 72 h treatment, the effect of the leaf extract in the colorectal carcinoma cell line increased significantly and exceeded that of the respective flower extracts. In the MCF-7 cell line, the leaf extract obtained from the wild plants was the most effective, while the extracts of flowers and leaves of cultivated plants showed comparable results. In contrast to the cancer cell lines, the control noncancer cells were significantly less affected and even at the highest tested extract concentrations, the cell viability was higher than 50% at the 24th h. With the prolongation of the treatment period to 72 h, the effect of the extracts on the normal cells increased; however, it was still lower than that found in all cancer cell lines tested. The extracts obtained from wild plants showed slightly higher antiproliferative activity than the cultivated plant extracts for all cell lines and treatment periods tested, with the exception of the HT-29 cell line where at 72 h, both the LCP and FCP extracts were more active compared to the LWP and FWP extracts.

#### 2.3.2. Fluorescent Microscopy

**Figure 5:**
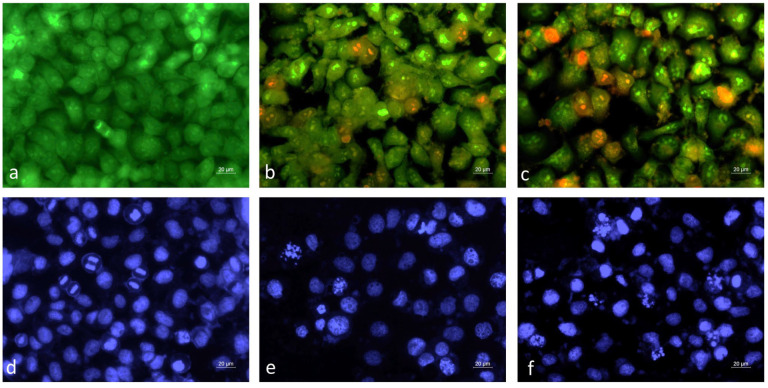
Fluorescence microscopy of cervical carcinoma cells HeLa treated with extracts of in vitro cultivated and wild-growing *M. vulgare* plants. (**a**,**d**) Untreated cells; (**b**,**e**) cells treated with extracts of cultivated plants; (**c**,**f**) cells treated with extracts of wild-growing plants. (**a**–**c**) Acridin orange/ethidium bromide staining; (**d**–**f**) 4′,6-diamino-2′-phenylindole (DAPI) staining.

Alterations in the cellular and nuclear morphology of HeLa cancer cells following treatment with extracts of micropropagated and wild-growing *M. vulgare* plants were analyzed and compared by fluorescent microscopy (Figure 5).

A monolayer of green-stained live cells was observed in the control untreated cell cultures labeled with acridin orange/ethidium bromide AO/EB (Figure 5a). Noticeable morphological changes in the cellular morphology of the HeLa cells treated with *M. vulgare* extracts were found (Figure 5b,c). In contrast to the control, cells actively undergoing mitosis were not found; the monolayer density was reduced and numerous early (intense green fluorescence) and late (red fluorescence) apoptotic cells were detected (Figure 5b,c). The cytopathological changes observed in the cells treated with extracts of in vitro cultivated and wild-growing *M. vulgare* plants were very similar. Further confirmation of the ability of the *M. vulgare* extract to trigger the apoptosis pathway in the cancer cells was provided by the 4′,6-diamino-2′-phenylindole (DAPI) nuclear staining. Cells treated with *M. vulgare* extracts showed characteristic apoptotic nuclear alterations, such as condensed chromatin, fragmented nuclei, and apoptotic bodies (Figure 5e,f).

#### 2.3.3. Cell Cycle Analysis

**Figure 6:**
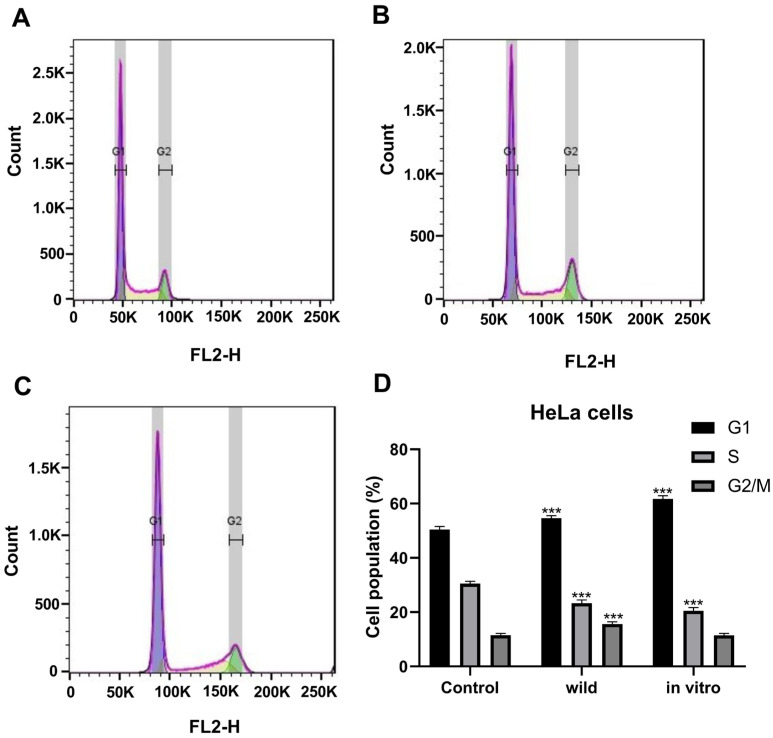
Effect of extracts of in vitro cultivated and wild-growing *M. vulgare* plants on the cell cycle progression of HeLa cervical carcinoma cells. Untreated cells (**A**); cells treated with extracts of wild-growing plants (**B**); cells treated with extracts of cultivated plants (**C**). Bar chart representing the distribution of the cells in the different cell cycle phases (**D**). The data are expressed as mean ± SD from three independent experiments; *** *p* < 0.001 indicates significant differences compared to the negative control.

Differences in cell distribution in the different phases of the cell cycle were observed after treatment with in vitro cultivated and wild-growing *M. vulgare* plants extracts. The results of the flow cytometry analysis are presented in Figure 6.

The treatment with both *M vulgare* extracts induced a significant increase in the G1 cell population and decreased the percentage of cells in the S phase. The cell population in the G2/M phase remained at the same level after treatment with the in vitro-grown plant extract (Figure 6C,D) but significantly increased upon treatment with the wild plant extract (Figure 6B,D). In the cells treated with the extract from the in vitro-grown plants, there was a strong accumulation of cells in G1, combined with a reduction in the cells in the S phase and no change in the cells at the G2/M phase. The obtained results could be interpreted as a G1 phase arrest of the cells (Figure 6C,D). In the cells treated with the wild plant extract, an accumulation of cells in two phases of the cell cycle (G1 + G2/M) was observed, which means that the treatment affected more than one checkpoint. A cell block was induced, affecting both G1 and the G2/M transition. The resulting effect on cell progression in the cell cycle was stronger for the wild plants than the effect obtained in cells treated with the extract from in vitro-grown plants, where mainly G1 arrest was observed.

## 3. Discussion

In the present study, a simple and effective micropropagation protocol using nodal segments from seedlings grown under in vitro conditions was developed. The in vitro culture was successfully initiated using seeds collected from naturally occurring wild plants.

The germination rate of the seeds in our study corresponded closely to the results reported by Mehalaine et al. [17], who demonstrated that seed germination under in vitro conditions significantly increased to 93.3% with the addition of 125 mg/L gibberellic acid (GA_3_), in contrast to a 23.3% germination rate for seeds germinated in a greenhouse. Similarly, Mehalaine et al. [18] reported that applying GA_3_ at concentrations ranging from 125 to 500 mg/L improved seed germination rates from 53.3% to 86.7% in ex vitro experiments.

In the current research, Murashige and Skoog (MS) basal medium was effectively used; however, cytokinin supplementation was necessary to induce and enhance shoot multiplication. Cytokinins are widely known to stimulate adventitious shoot formation by promoting cell division, enhancing DNA synthesis, and regulating the cell cycle [19]. Each of the tested cytokinins—6-benzylaminopurine (BAP), kinetin, and zeatin—had a statistically significant effect on the number of shoots per explant. The highest shoot multiplication rate of *M. vulgare* was achieved with 1.0 mg/L kinetin.

Previous studies have demonstrated that varying concentrations and types of cytokinins and auxins in the culture medium significantly influence the growth and morphogenesis of *M. vulgare* tissues in vitro. Abdallah et al. [12] investigated the effects of different culture media—MS (Murashige and Skoog, 1962) [16], B5 (Gamborg et al., 1968) [20], and NN (Nitsch and Nitsch, 1969) [21]—on bud formation, bud size, and leaf number. Similarly to our findings, the authors reported prolific shoot formation when MS medium was supplemented with 1.0 mg/L kinetin, concluding that MS medium was the most suitable for multiple shoot formation. Unlike our protocol, however, Abdallah et al. [12] included α-naphthaleneacetic acid in their medium, whereas in our study, indole-3-acetic acid was used. This resulted in a higher number of buds per explant (4.00 ± 0.22). Knöss et al. [11] reported spontaneous bud formation in callus cultures grown on hormone-free MS medium. Mehalaine and Chenchouni [13] observed shoot proliferation in only 33.3% to 36.7% of explants when using the same cytokinin–auxin combination as in our study (kinetin and IAA), but at significantly higher concentrations—from 2.5 mg/L up to 5.0 mg/L. These findings highlight the efficiency of our lower-concentration cytokinin–auxin protocol for inducing multiple shoots in *M. vulgare*. As there are no data in the literature regarding the effects of different culture media and combinations of growth regulators (such as auxins and cytokinins) on the levels of phenolics, flavonoids, and other components with anticancer properties in *M. vulgare*, our micropropagation protocol mostly aimed to provide the most favorable conditions for plant growth and development, ensuring mass production of large quantities of plants in a short period of time with constant reproducible content of active compounds when following the chosen micropropagation protocol.

Polyphenols, a diverse group of plant-derived compounds, are classified into four major categories—flavonoids, phenolic acids, stilbenes, and lignans—based on their chemical structure. Polyphenols, including flavonoids, are largely known for having a beneficial effect on heart and brain health, on diabetes prevention, and for cancer risk reduction [22]. The anticancer potential of natural polyphenols is predominantly ascribed to their strong antioxidant and anti-inflammatory properties, alongside their capacity to influence key molecular targets and signaling cascades implicated in various cellular and physiological processes, including cell viability, cell differentiation and proliferation, cell migration, angiogenesis, hormonal regulation, activation of enzymes involved in eliminating reactive oxygen species, and modulation of immune function [23]. Our results show that, with the exception of polyphenols in flowers (where polyphenol content is comparable), the total polyphenol as well as flavonoid content is consistently higher in the *Marrubium* extracts from the cultivated plants in comparison to the extracts from the respective counterparts (i.e., flowers, leaves, and stems) of the wild plants with flavonoid content completely missing in the flowers and stems of wild plants. This extends also to the ORAC and HORAC activities defined in our study for every particular part of both cultivated and wild *Marrubium* plants, with both ORAC and HORAC activities being more than four times lower for the flowers of wild plants compared to the flowers of cultivated plants. No comparison could be made with other studies regarding the total polyphenol content and antioxidant activity with respect to the difference between wild and in vitro obtained and cultivated plants as, to our knowledge, no other study except ours characterizes those properties in in vitro cultivated plants. The same refers to those parameters in the different parts of wild plants as data for the polyphenol content and antioxidant activity of extracts from the whole plants are mostly available with some studies considering extracts from the leaves as well. If we would to, anyway, compare phenolic and flavonoid content with data in the literature, for instance, Guedri Mkaddem et al. [24] reported that the total polyphenol content of leaves from eight natural populations of *M. vulgare* in Tunisia ranged from 20.8 to 44.7 mg GAE/g DW, which is close to, and slightly higher than, the values obtained in our study. Other publications present data for total polyphenol and flavonoid contents determined in liquid [15] or dried extracts [25,26]. Although a precise comparison is hindered by methodological differences, recalculating these results based on the reported extraction yields (typically 10–20%) indicates that the polyphenol content on a plant material basis lies between 617 and 1243 mg GAE/100 g DW [27,28], which is comparable to our results for stems and flowers, but lower than that determined for leaves. Regarding flavonoids, Guedri Mkaddem et al. [24] found that the total flavonoid content of *M. vulgare* leaves varied from 891 to 3748 mg RE/100 g DW, values considerably higher than those recorded in our investigation. Additional studies reporting flavonoid contents in liquid [15] or dried extracts [25,26] express their results per gram of extract rather than per gram of plant material. When normalized to the dry plant basis using the reported extraction yields (10–20%), the corresponding total flavonoid content is estimated to be in the range of 430–1150 mg RE/100 g DW [28], which is comparable to or slightly lower than our results.

The total polyphenols and flavonoid content, which is higher in leaves and flowers compared to stems in both wild and cultivated plants (exceptions are the flowers of wild plants which lack any flavonoid content), certainly translates to their anticancer, antiproliferative activity.

Thus, at first glance, comparing the three different parts of both wild and cultivated plants, leaves, and flowers are more effective in their anticancer, antiproliferative effect than stems. Second of all, treatment for 72 h is more effective than treatment for 24 h in all cases of leaves, flowers, and stems for both cultivated and wild plants, possibly reflecting the need for a longer exposure for the active compounds to take an effect. Third, for the more therapeutically active parts of the plants, flowers, and leaves, at the more effective time of treatment, 72 h, cultivated plants compared to the wild ones are less toxic based on the IC_50_s of the extracts on the noncancer, fibroblast control cell line (535.2 µg/mL for LWP vs. 669.3 µg/mL for LCP and 596.0 µg/mL for FWP vs. 694.2 µg/mL for FCP).

Considering more closely the three cancer cell lines and the antiproliferative potential of the different extracts, based on their inhibitory IC_50_ concentrations determined by the MTT assay at 72 h, it is obvious that *M. vulgare* is the most effective against cervical cancer, as the antiproliferative capacity is highest for the HeLa cell line and is in the order FWP > FCP > LWP > SCP~LCP, with the IC_50_ for the flowers of wild plants being as low as 42.0 µg/mL and the IC_50_ for the stems and leaves of cultivated plants being as high as 306.0 µg/mL and 307.0 µg/mL, respectively. Next in line comes colorectal cancer with extracts of cultivated plants (leaves and flowers) being most efficient against it, followed closely by mammary carcinoma (IC_50LCP_ HT-29 (307.5 µg/mL) < IC_50FCP_ HT-29 (330.1 µg/mL) < IC_50FWP_ MCF-7 (342.8 µg/mL) < IC_50FCP_ MCF-7 (361.4 µg/mL) < IC_50LCP_ MCF-7 (375.6 µg/mL) < IC_50LWP_ HT-29 (377.1 µg/mL) < remaining extracts with IC_50_ above 400 µg/mL). It should be noted that standing out from the above classification is the leaves of wild plants with quite high antiproliferative potential against human mammary carcinoma (IC_50LWP_ = 234.0 µg/mL).

In summary, it could be concluded that while both leaves and flowers are useful against all three types of tumors, as judged by their antiproliferative efficacy against the studied representative cancer cell lines, the extracts of flowers (followed closely by leaves) are most efficient on cervical cancer (HeLa) (42.0 µg/mL for FWP and 168.0 µg/mL for FCP, followed by 235.2 µg/mL for LWP and 307.0 µg/mL for LCP) and the extracts of wild leaves (followed closely by flowers of both cultivated and wild plants) on mammary carcinoma (234.0 µg/mL for LWP, followed by 342.8 µg/mL for FWP and 361.4 µg/mL for FCP and 375.6 µg/mL for LCP).

Considering the polyphenol and flavonoid content of flowers, it could be seen that they are neither as rich in polyphenols and flavonoids as the leaves, neither is their antioxidant activity as high. Actually the flowers of wild plants which are the most effective against the HeLa cell line completely lack flavonoids and their HORAC and ORAC activities are lower than that even of stems. However, except for polyphenols and flavonoids, *M. vulgare* produces essential oils with volatile monoterpenes and sesquiterpenes and their derivatives such as α-pinene, camphene, β-citronellol, germacrene-D, γ-eudesmol, and others [7,28]. Cytotoxicity assay has shown the capability of *M. vulgare* essential oil to inhibit the proliferation of HeLa cell lines [7]. Additionally, present in the plant are non-volatile monoterpenes such as marrubic acid and sacranoside A, a sesquiterpene lactone like vulgarin, a phytosterol like β-sitosterol, triterpenoids like lupeol and oleanolic acid, and a great variety of diterpenoids, the most abundant of which is marrubiin [28]. Research highlights marrubiin’s anti-inflammatory and immunomodulatory effects, which are mechanistically linked to its cytotoxic potential in inflammatory models [29]. Although no direct evidence for an anticancer cytotoxic effect of marrubiin has been given so far in the literature, it is a labdane-type diterpene, a class known for its anticancer properties. Other labdanes like coronarin D, andrographolide, and sclareol have long shown potent anticancer effects via apoptosis, NF-κB inhibition, and COX modulation [30]. Our study shows that indeed marrubiin is the most abundant in the flowers of wild and cultivated plants, the extracts of which are most effective against the HeLa cancer cell line. Regarding the anticancer activity against HeLa, it is important to highlight that for lupeol, β-sitosterol, and oleanolic acid and its derivatives, this activity is direct and well-documented with some of the mechanisms of the anticancer effect being ROS generation, Bax/Bcl-2 modulation, mitochondrial disruption, S-phase cell cycle arrest, ACSL4 (acyl-CoA synthase long chain family member 4) ferroptosis signaling pathway changes, and the induction of apoptosis [31,32,33,34].

Regarding the antiproliferative activity against the MCF-7 mammary carcinoma cell line, which is the highest for wild leaves, it is not unexpected as leaves have the highest content of polyphenols, flavonoids, and ORAC and HORAC antioxidant activities amongst all studied parts of both wild and cultivated plants. The polyphenol content of leaves is almost twice as high as that of flowers and even much higher than that of wild stems. Indeed, polyphenols and, in particular, phenolic acids have long been recruited in the fight against breast cancer. Scientific data show antiproliferative and cytotoxic effects of ferulic, caffeic, gallic, and p-coumaric acids against the MCF-7 cell line and anticancer activity against mammary adenocarcinoma [35,36,37]. Concerning flavonoids, acacetin, apigenin, and acacetin-7-rhamnoside directly isolated from *M. vulgare*, they showed very high cytotoxic activity against MCF-7 with ED_50_ ≤ 20 μg/mL [3]. Of course we cannot exclude the presence of a particular bioactive compound that occurs in the extracts of wild plant leaves and is missing or in a smaller amount in the extracts of cultivated plant leaves, since it could explain the higher antiproliferative potential against MCF-7 particularly of wild plant leaves.

Overall, extracts from wild-growing plants demonstrate slightly higher antiproliferative activity than their cultivated counterparts, indicating that environmental factors may influence the biosynthesis and accumulation of bioactive metabolites. Exceptions are observed in HT-29 cells at 72 h, where leaf and flower extracts from cultivated plants (LCP, FCP) surpass those from wild plants (LWP, FWP). This suggests that cultivation conditions may favor the production of compounds selectively effective against colorectal carcinoma cells. The observed differences between wild and cultivated material highlights the need to optimize in vitro culture conditions to maximize the therapeutic potential of *M. vulgare*.

Our observation of a pronounced antiproliferative effect exerted by extracts from various aerial parts of *M. vulgare* on human cancer cell lines—while exhibiting minimal cytotoxicity toward non-malignant cells—corroborates the existing literature that highlights the selective in vitro anticancer activity of diverse *M. vulgare* extracts. The nonhydrolyzed phenolic acid fraction obtained from methanolic extracts showed cytotoxic activity toward the melanoma cell line A375, while normal human skin fibroblasts BJ were not affected [38]. Similarly, after treatment with *M. vulgare* ethanolic extracts, a concentration-dependent reduction in viability was detected in melanoma (B16) and glioma (U251) cells, but not in peripheral blood mononuclear cells [39]. The methanolic extract of the plant and its fractions showed significant cytotoxicity against the human cancer cell lines MCF-7, HT29, and SW480 and had a weak impact on the non-cancerous cell line MRC5 [40]. The relative resistance of non-tumorigenic cells suggests that *M. vulgare* extracts preferentially target transformed cells, possibly due to differences in proliferation rates, the cell redox status, or the sensitivity of signaling pathways regulating apoptosis and cell cycle progression. In addition, numerous reports have indicated the antiproliferative activity of the plant extracts in different human cancer cell lines of various tissue origin, including cervical carcinoma (HeLa), mammary carcinoma (MCF-7 and MDA-MB-231), lung carcinoma (A549), liver carcinoma (HepG2), colorectal carcinoma (HT-29 and SW480), glioblastoma (U87, LN229, T98G and U251), and melanoma (B16 and A375) [7,38,39,40,41,42]. Most of these studies examine alcoholic extracts, while data regarding the biological activity of aqueous extracts are limited. For this reason, the present study focused on the assessment of the antiproliferative activity of aqueous extracts of *M. vulgare*. Moreover, the activity of extracts obtained from different anatomical parts of the plant was considered.

Referring to the mechanism of the anticancer effect of the *M. vulgare* extract, it has been studied on the HeLa cell line, with HeLa being the most sensitive of the cancer cell lines to the plant extracts. Microscopic and flow cytometric analyses provided insights into the mechanisms underlying the antiproliferative effects of the extracts. Fluorescence microscopy revealed typical apoptotic alterations in the HeLa cells, including nuclear condensation, fragmentation, and apoptotic body formation. AO/EB staining confirmed the presence of both early and late apoptotic cells, with a marked reduction in mitotic activity, supporting apoptosis induction as a key mechanism of action.

Flow cytometry further demonstrated that *M. vulgare* extracts interfere with cell cycle progression. Extracts from cultivated plants predominantly induced G1 phase arrest, accompanied by a reduction in the S-phase cells, consistent with the inhibition of DNA synthesis. By contrast, extracts from wild plants exerted a stronger and broader effect, causing the accumulation of cells in both G1 and G2/M phases, suggesting interference with multiple checkpoints. This dual arrest points to a more potent disruption of the cell cycle, which may explain the slightly higher antiproliferative activity of wild extracts compared to cultivated ones.

The present study confirms and extends prior evidence that *M. vulgare* extracts exert selective antiproliferative effects on cancer cells and act through cell cycle disruption and induction of apoptosis. In a previous more detailed mechanistic study on *M. vulgare* ethanolic extracts, it was found that the anticancer effects were associated with mitochondrial depolarization, the activation of caspase-9 and -3, PARP cleavage, and the up-regulation of pro-apoptotic genes (Pten, Bak1, Apaf1, Puma) together with the down-regulation of survivin and XIAP [36]. Those molecular signatures are consistent with the apoptotic morphology detected in our study and suggest that *M. vulgare* extracts may trigger apoptosis following cell cycle perturbation.

Taken together, the anticancer activity of the different parts of the plant flower extracts displayed particularly strong activity in HeLa cells, whereas leaf extracts showed superior efficacy in MCF-7 and HT-29 cells. Concerning MCF-7 cells, leaf extracts from the wild plants were the more active, whereas regarding HT-29 cells, the leaf extracts from the cultivated plants showed a higher antiproliferative activity than the leaf extracts from the wild plants. Collectively, our results indicate that both leaf and flower tissues represent valuable sources of anticancer agents, but their activity is context-dependent, influenced by the cancer cell type.

Regarding the sensitivity of the different types of cancer, as judged by the effect of the *M. vulgare* extracts on the different cancer cell lines, the tested cell lines exhibited distinct sensitivity profiles. HeLa cells were the most responsive, as mentioned above, particularly to flower extracts, where IC_50_ values reached as low as 42.0 µg/mL after 72 h. MCF-7 breast cancer cells also responded strongly, particularly to wild leaf and flower extracts, while HT-29 colorectal carcinoma cells were more sensitive to cultivated plant extracts. The non-cancerous cells BALB/3T3 showed the least sensitivity to *M. vulgare* extracts, possibly reflecting differences in the proliferation rate, metabolic capacity, membrane transport systems, efflux mechanisms, and signaling pathways of the individual cell types.

## 4. Materials and Methods

### 4.1. In Vitro Culture

#### 4.1.1. Initial Plant Material

The seeds for in vitro experiments were collected from the district of Teshel (West Rodopa Mountain, 989 m; 41.672106° N 24.351968° E).

#### 4.1.2. Sterilization of Plant Material

*M. vulgare* seeds were surface disinfected through an immersion in a 70% ethyl alcohol solution for 2 min, followed by soaking in a 50% commercial bleach solution (containing 4.85% active chlorine) for 10 min. Next, the seeds were rinsed threefold with autoclaved water in a laminar airflow cabinet to remove bleach residues. The aseptic seeds were cultured on basal Murashige and Skoog [16] medium (containing 30 g/L sucrose) for germination.

#### 4.1.3. Media Composition for in Vitro Micropropagation

For in vitro shoot multiplication, the obtained seedlings were divided into small segments between 0.5 and 1 cm in length. Eight different MS-based media, modified for shoot formation and development, were examined. The media contained one cytokinin, zeatin (Z) (a mixed isomere) (Sigma-Aldrich Inc., Burlington, MA, USA), 6-benzylaminopurine (BAP), or kinetin (K), and in some cases, it was combined with one of three auxins: indole-3-butyric acid (IBA), α-naphthalene acetic acid (NAA), or indole-3-acetic acid (IAA) (Duchefa Biochemie B.V, Haarlem, The Netherlands) (Table 1). The composition of modified MS media was as follows: K_1_ (kinetin 1.0 mg/L); K_1.5_ (kinetin 1.5 mg/L); K_2_ (kinetin 2.0 mg/L); K_1_IAA_0.1_ (kinetin 1.0 mg/L and IAA 0.1 mg/L); Z_1_IAA_0.1_ (zeatin 1.0 mg/L and IAA 0.1 mg/L); BAP_1_IAA_0.1_ (BAP 1.0 mg/L and IAA 0.1 mg/L); K_1_NAA_0.1_ (kinetin 1.0 mg/L and NAA 0.1 mg/L); K_2_IBA_0.2_ (kinetin 2.0 mg/L and IBA 0.2 mg/L). The explants were repeatedly transferred to fresh medium in order to produce numerous shoots. The multiplication efficiency, the number of shoots per explant, and shoot height were recorded after 4 weeks of culturing.

#### 4.1.4. In Vitro Rooting and Acclimatization of Obtained Plants

In vitro-raised shoots with approximately 1.5–2 cm of length were transferred to half strength MS medium supplemented with different concentrations of IBA and IAA. The rooting frequency, the number of roots per shoot, and the rooting length were measured after four weeks of culture.

The rooted in vitro plants were gently rinsed with tap water and then transferred to plastic pots (8 cm in diameter). The pots were filled with a substrate composed of soil, peat, perlite, and sand in a ratio of 2:1:1:1.

The pots were shielded by clear plastic boxes for two weeks to guarantee high relative humidity. Additional culturing parameters were 50 μM m^−2^ s^−1^ light, 25 °C, and a 16/8 photoperiod. Survival rate was recorded after five weeks of culture. Final acclimatization was carried out in the Gorna baniya field plots (Sofia district, 800 m altitude, April 2024).

#### 4.1.5. Conditions for In Vitro Cultures

The in vitro cultures were maintained in a growth room under artificial lighting provided by FL^-40^ W^−1^ fluorescent lamps (Svetlina Ltd., Stara Zagora, Bulgaria), with a 16 h light/8 h dark photoperiod, at a temperature range of 18–21 °C and a light intensity of 40 μmol m^−2^ s^−1^.

### 4.2. Plant Material Extraction and Analysis

#### 4.2.1. Plant Material

For phytochemical and anti-tumor activity assessments, aerial parts from both wild plants and in vitro cultivated specimens grown in an experimental field were harvested. The plant material was collected during the summer of 2024 (July–August), then air-dried in the shade at ambient temperature. Following drying, leaves, flowers, and stems were separated, placed in paper bags, and stored at room temperature for analysis.

#### 4.2.2. Extraction of Polyphenols and Flavonoids

For the determination of total polyphenols, total flavonoids, and antioxidant activity, plant materials were powdered with a laboratory mill (Optimum RK-0150, Warsaw, Al. Witosa 31/22, Poland). Next, 2 g of the powder was mixed with 40 mL 65% ethanol in an extraction tube. The samples were extracted for 1 h at room temperature on a magnetic stirrer (Multistirrer 6, Velp Scientifica Srl, Usmate, Italy). Subsequently they were centrifuged (6000× *g*) and supernatants removed. Clear supernatants were used for the analysis of total polyphenols and flavonoids and antioxidant activity.

#### 4.2.3. Determination of Total Polyphenol and Total Flavonoid Content

Total polyphenol content was determined using the method of Singleton and Rossi, 1965 [43] with gallic acid as a calibration standard. Extracts (0.1 mL) or gallic acid standard solutions were mixed in a test tube with 3.1 mL of deionized water, 0.2 mL of Folin–Ciocalteu phenol reagents, and 0.6 mL of 20% sodium carbonate, and incubated for 5 min at 60 °C. Next test tubes were transferred to an ice water bath for 5 min and the absorbance was measured at 765 nm. The results were expressed in mg Gallic acid equivalents (GAE) per 100 g dry weight (DW) ± SD.

The total flavonoid content was determined according to Chang et al. [44] using an AlCl_3_ reagent and a calibration curve built with rutin (10–200 mg/L) as a standard compound. Briefly, 0.4 mL of the extract or standard (rutin) solution are mixed with 2.24 mL of distilled water, 1.2 mL of absolute ethanol, 0.08 mL of 1 M potassium acetate solution, and 0.08 mL of 10% aluminum chloride solution. A blank sample containing 2.32 mL of distilled water, 0.4 mL of the sample, 1.2 mL of absolute ethanol, and 0.08 mL of 1 M potassium acetate but no aluminum chloride was also prepared individually for each analyzed sample. Samples were vortexed to ensure homogeneity and incubated at room temperature for 30 min. After incubation, the mixtures were transferred to glass UV cuvettes, and absorbance was measured using a spectrophotometer (Dynamica Halo RB-10, Dynamica Scientific Ltd., Livingston, UK) at a wavelength of 415 nm. For each sample, the corresponding blank was measured first. The results are expressed as mg rutin equivalents (RE) per 100 g DW ± SD.

#### 4.2.4. Determination of Marrubiin Content

Marrubiin content in the extract was determined using a slightly modified method based on the European Pharmacopoeia (Ph Eur) monograph 1835 [45]. The procedure is given in detail in Lazarova et al. [46]. In short, chromatographic evaluation was performed utilizing a Nexera-i LC2040C Plus UHPLC system (Shimadzu Corporation, Kyoto, Japan), integrated with a UV-VIS detector and a binary pumping module. Separation was achieved on a Poroshell 120 EC-C18 column (3 mm × 100 mm, 2.7 μm), maintained at 26 °C. The mobile phase was delivered at a flow rate of 1.5 mL/min, and sample injections were set at 20 μL. Detection of analyte derivatives was carried out at 330 nm. The elution system comprised solvent A (acetonitrile) and solvent B (0.5 mL phosphoric acid diluted to 1000 mL with deionized water), with a programmed gradient applied according to the established protocol.

#### 4.2.5. Antioxidant Activity Assays

Oxygen radical absorbance capacity (ORAC) was measured according to the methodology of Ou et al. [47], with details reported by Denev et al. [48].

Briefly, solutions of AAPH, FL, and Trolox were prepared in a phosphate buffer (75 mmol/l, pH 7.4). Samples were diluted in the phosphate buffer as well. The reaction mixture (total volume 200 μL) contained FL (170 μL, final concentration 5.36 × 10^−8^ mol/L), AAPH (20 μL, final concentration 51.51 mmol/L), and sample—10 μL. The FL solution and sample were incubated at 37 °C for 20 min directly in a microplate reader FLUOstar OPTIMA (BMG Labtech, Ortenberg, Germany), and AAPH (dissolved in buffer at 37 °C) was added. The mixture was incubated for 30 s before the initial fluorescence was measured. After that, the fluorescence readings were taken at the end of every cycle (1 min) after shaking. For the blank, 10 μL of phosphate buffer was used instead of the extract. Trolox solutions (6.25; 12.5; 25; and 50 μmol/L) were used for defining the standard curve. ORAC was measured using a FLUOstar OPTIMA plate reader (BMG Labtech, Ortenberg, Germany), with an excitation wavelength of 485 nm and emission wave length of 520 nm used. ORAC values were expressed in µmol TE per gram dry plant material ± SD.

Hydroxyl radical averting capacity (HORAC) was measured based on Ou et al. [49]. Briefly, a hydrogen peroxide solution of 0.55 M was prepared in distilled water. Then, 4.6 mM Co(II) was prepared as follows: 15.7 mg of CoF2.4H_2_O and 20 mg of picolinic acid were dissolved in 20 mL of distilled water. FL—170 μL (60 nM, final concentration) and 10 µL of sample were incubated in 37 °C for 10 min directly in the FLUOstar plate reader. After incubation, 10 μL H_2_O_2_ (27.5 mM, final concentration) and 10 μL Co(II) (230 µM final concentration) solutions were added subsequently. The initial fluorescence was measured, after which the readings were taken every minute after shaking. For the blank sample, phosphate-buffer solution was used. Then, 100, 200, 600, 800, and 1000 μM gallic acid solutions (in phosphate buffer 75 mM, pH = 7.4) were used for building the standard curve. Measurements were performed on a FLUOstar OPTIMA fluorometer (BMG Labtech, Offenburg, Germany). The excitation wavelength of 485 nm and emission wavelength of 520 nm were used. The results were expressed in micromole gallic acid equivalents (µmol GAE) per gram dry plant material ± SD.

#### 4.2.6. Preparation of Freeze-Dried Extract for Anti-Tumor Activity Analysis

For testing anti-tumor activity, freeze-died extracts were prepared by the following procedure: 5 g of plant material powder was added to 200 mL water (90 °C) and incubated for 15 min. The slurry was centrifuged (6000× *g*) and supernatant was collected and freeze-dried for 96 h in an Alpha 1–4 LD plus laboratory freeze drier (Martin Christ Gefriertrocknungsanlagen GmbH, Osterode am Harz, Germany).

### 4.3. Anti-Tumor Activity

#### 4.3.1. Cell Lines

Cell lines of murine embryo fibroblasts BALB/3T3 (ATCC CCL-163), human cervical carcinoma HeLa (ATCC CCL-2), human colorectal adenocarcinoma HT-29 (ATCC HTB-38), and human mammary carcinoma MCF-7 (ATCC HTB-22) were purchased from the American Type Culture Collection (Rockville, MD, USA). All cell lines were cultured in DMEM medium, supplemented with 10% fetal bovine serum, 100 U/mL penicillin, and 100 μg/mL streptomycin, at 37 °C in a humidified 5% CO_2_ incubator (HEPA class 100, Thermo Scientific, Marietta, OH, USA). Confluent cell monolayers were trypsinized (mixture of 0.05% trypsin–0.02% ethylendiaminotetraacetic acid) and cells were used at the exponentially growing phase.

#### 4.3.2. Assessment of Cell Viability

The effects of the *M. vulgare* extracts on cancer cell viability were evaluated using the standard colorimetric MTT assay. Non-tumorigenic BALB/3T3 cells were used as a reference cell line to assess the selectivity of the antiproliferative effects towards cancer cells. After cell dissociation using a 0.25% Trypsin-EDTA solution and counting in a hemocytometer, cell suspensions were adjusted to a density of 1 × 104 cells/mL and plated in 96-well plates. The cells were incubated overnight at 37 °C in a humidified atmosphere containing 5% CO_2_. The next day, *M. vulgare* extracts obtained from the different aerial parts of in vitro cultivated and wild-growing *M. vulgare* plants were added to the cells at concentrations ranging from 125 μg/mL to 1000 μg/mL. Untreated cells cultivated under the same conditions were used as controls. Each concentration of the extracts was tested in five replicates. After being cultured in the presence of the extracts for 24 and 72 h, the cells were washed with PBS, and 100 μL MTT solution in DMEM (5 mg/mL) was added to each well. The cell cultures were then incubated at 37 °C for 3 h, with the supernatants discarded and 100 μL of lysing DMSO/ethanol solution (in 1:1 ratio) added to dissolve the obtained purple formazan crystals. The absorbance of the samples was measured at 570 nm using an ELISA plate reader (TECAN, SunriseTM, Grodig/Salzburg, Austria). The viability of the treated cell cultures was presented as a percentage of the respective untreated control.

#### 4.3.3. Analysis of Cell Death

The ability of the *M. vulgare* extracts to induce cancer cell death was examined after live/dead staining with the fluorescent dyes acridine orange (AO) and ethidium bromide (EtBr). HeLa cells were plated at a density of 1 × 10^5^ cells/well on sterile glass coverslips, placed in a 24-well cell culture plate, and incubated overnight. The next day, the cells were treated with FWP and FCP *M. vulgare* extracts applied at concentrations approximating half of the IC_50_ values determined by the MTT assay at 24 h (250 μg/mL and 350 μg/mL, respectively). Untreated cells, cultivated in the same conditions but only in growth medium, served as negative controls. After 24 h of treatment, the coverslips were removed, rinsed with phosphate-buffered saline (PBS), and equal volumes of AO (10 μg/mL in PBS) and EtBr (10 μg/mL in PBS) were added to the cells. Fluorescently stained cells were mounted on glass slides and immediately examined using a fluorescence microscope Leica (DM 5000B, Wetzlar, Germany) before the fluorescent color started to fade.

#### 4.3.4. Nuclear Morphology Analysis

The nuclear morphology of HeLa cells treated with the FWP and FCP *M. vulgare* extracts was also investigated by staining with the DNA-binding dye 4′,6-diamino-2′-phenylindole (DAPI). Cells were cultivated and treated as described in the previous section. After incubation, the glass coverslips were washed twice with phosphate-buffered saline (PBS) to remove detached cells and cell debris. DAPI staining was performed after fixation with ice-cold methanol according to a procedure recommended in the manufacturer’s protocol. Samples of treated and untreated control cells were mounted on microscope slides and stored in the dark until examination with the fluorescence Leica microscope (DM 5000B, Wetzlar, Germany) was performed.

#### 4.3.5. Cell Cycle Flow Cytometry Analysis

The effect from the treatment with the *M. vulgare* extract on the cell cycle progression of cervical carcinoma HeLa cells was evaluated by flow cytometry. Cells were seeded in 6-well plates, incubated for 24 h, and treated with the *M. vulgare* extracts at the same concentrations as those used for the fluorescent microscopy studies. Untreated HeLa cells were used as controls. After 24 h of treatment, the cells were detached with Trypsin-EDTA solution (Sigma-Aldrich) and washed twice with cold phosphate-buffered saline (PBS) by centrifugation at 1000 rpm for 10 min. Cell pellets were collected and fixed with 70% ice-cold ethanol, added dropwise while vortexing. Cells were stored overnight at −20°C. The fixed cells were washed with PBS and treated with 20 µg/mL RNase A (Roche Diagnostics GmbH, Mannheim, Germany) for half an hour. After staining with propidium iodide (20 µg/mL), cell populations at different stages of the cell cycle were analyzed by a flow cytometer (Becton Dickinson, BD Biosciences, San Jose, CA, USA). The percentages of cells in different cell cycle phases (G1, S, and G2-M) were determined using FlowJo™ v10.8 software (BD Biosciences, San Jose, CA, USA). Data are presented as the mean ± standard error of the mean of three replicates.

### 4.4. Statistical Analysis

The results were expressed as mean values ± standard error of the mean. Data were analyzed with the use of one-way analysis of variance (ANOVA) GraphPad Prism 9.0, followed by Bonferroni post hoc comparison test. Differences were considered significant at (SEM) *p* < 0.05.

## 5. Conclusions

This study presents, for the first time, a comprehensive comparison between water extracts derived from in vitro cultivated and wild-growing *M. vulgare* plants, linking their polyphenols and flavonoid content with antioxidant and anticancer properties. An efficient micropropagation protocol was successfully developed, providing a sustainable source of biologically active plant material and addressing the limited availability of wild populations.

The results in the present study reveal that *M. vulgare* extracts, particularly from leaves and flowers, exhibit significant and selective antiproliferative activity against human cervical (HeLa), breast (MCF-7), and colorectal (HT-29) cancer cell lines, with minimal cytotoxicity towards normal cells. The anticancer mechanisms involve apoptosis induction and cell cycle arrest, with cultivated plant extracts primarily causing G1 arrest and wild plant extracts affecting both the G1 and G2/M phases. Thus, our findings are the first to demonstrate the anticancer potential of both wild and in vitro cultivated *M. vulgare* plants, prompting their further use in future anticancer therapeutic applications. The elevated flavonoid levels in in vitro cultivated plants compared to wild plants, together with enhanced antioxidant capacity, indicate the strong potential of in vitro cultivated plants in antioxidant and cytoprotective formulations aimed at mitigating oxidative stress-related pathologies such as cardiovascular, neurodegenerative, and metabolic diseases.

## Figures and Tables

**Table 1:** Effect of different plant growth regulators on *M. vulgare* shoot multiplication.

Plant Growth Regulators [mg/L]	New Shoot Formation [%]	Number of Shoot per Explant	Shoot Height [cm]
K_1_	60	3.08 ± 0.33 ^c^	2.00 ± 0.20 ^cd^
K_1.5_	80	2.75 ± 0.26 ^c^	1.60 ± 0.16 ^b^
K_2_	95	3.00 ± 0.24 ^c^	1.80 ± 0.16 ^bc^
K_1_IAA_0.1_	90	4.00 ± 0.22 ^d^	3.70 ± 0.24 ^f^
Z_1_IAA_0.1_	70	2.35 ± 0.13 ^b^	3.5 ± 0.22 ^f^
BAP_1_IAA_0.1_	80	1.93 ± 0.19 ^a^	2.70 ± 0.23 ^e^
K_1_NAA_0.1_	80	2.81 ± 0.20 ^c^	2.21 ± 0.19 ^d^
K_2_IBA_0.2_	40	2.25 ± 0.16 ^ab^	1.01 ± 0.10 ^a^
LSD	-	0.37	0.33

Legend: K—kinetin, IAA—indole-3-acetic acid, Z—zeatin, BAP—6-benzylaminopurine, NAA—α-naphthalene acetic acid, IBA—indole-3-butyric acid. The data are presented as mean values of 40 buds for the mean ± standard error (SE). Different letters indicate the significance of the analysis, assessed by Fisher’s LSD test (*p* ≤ 0.05) after applying a one-way ANOVA. The letters “a”, “b”, “c”, “d”, “e”, and “f” next to the values in the table refer to the statistical groupings as a result of the LSD test. When the two values share the same letter, no significant difference is demonstrated; a significant difference is denoted by different letters next to the value.

**Table 2:** Rooting in vitro of *M. vulgare* (20 days of cultivation).

Nutrient Medium	Root Induction [%]	Number of Roots Per Explants	Root Length [cm]
IBA_0.1_	50	2.8 ± 0.24 ^a^	1.5 ± 0.15 ^b^
IBA_1.0_	70	6.0 ± 0.23 ^b^	1.0 ± 0.09 ^a^
IBA_2.0_ IAA_0.2_	90	6.4 ± 0.22 ^b^	1.3 ± 0.13 ^b^
LSD		0.45	0.25

Legend: IBA—indole-3-butyric acid, IAA—indole-3-acetic acid. The data are presented as mean values of 40 buds for the mean ± standard error (SE). Different letters indicate the significance of the analysis, assessed by Fisher’s LSD test (*p* ≤ 0.05) after applying a one-way ANOVA. The letters “a” and “b” next to the values in the table refer to the statistical groupings as a result of the LSD test. When the two values share the same letter, no significant difference is demonstrated between them; a significant difference is denoted by different letters next to the values.

**Table 3:** Total polyphenol and flavonoid content and antioxidant activity of in vitro obtained field-grown and cultivated *M. vulgare* plants and wild-growing plants.

Sample	Total Polyphenols, mg GAE/100 g DW	Total Flavonoids, mg RE/100 g DW	ORAC, µmol TE/g DW	HORAC, µmol GAE/g DW
FCP	1205.9 ± 10.0 ^a^	215.2 ± 9.0 ^b^	297.8 ± 12.4 ^a^	118.0 ± 5.2 ^a^
FWP	1274.5 ± 5.0 ^b^	n.d.	67.8 ± 1.2 ^b^	28.8 ± 1.9 ^b^
LCP	2100.5 ± 3.3 ^c^	325.4 ± 15.9 ^d^	518.6 ± 20.8 ^c^	194.5 ± 11.3 ^c^
LWP	1996.1 ± 7.7 ^d^	236.5 ± 10.0 ^c^	492.9 ± 2.4 ^d^	193.6 ± 15.7 ^d^
SCP	1030.1 ± 3.4 ^e^	68.2 ± 3.9 ^a^	254.4 ± 20.6 ^e^	108.1 ± 6.4 ^e^
SWP	481.6 ± 5.6 ^f^	n.d	118.9 ± 5.8 ^f^	50.1 ± 3.6 ^e^
LSD	6.50	19.95	13.61	8.99

Legend: GAE—gallic acid equivalents, DW—dry weight, RE—rutin equivalents, TE—Trolox equivalents, ORAC—oxygen radical absorbance capacity, hydroxyl radical averting capacity—HORAC; FCP (flowers of in vitro cultivated plants); FWP (flowers of wild plants); LCP (leaves of in vitro cultivated plants); LWP (leaves of wild plants); SCP (stems of in vitro cultivated plants); SWP (stems of wild plants); n.d.—not detected. Different letters indicate the significance of the analysis, assessed by a post hoc Fisher’s LSD test (*p* ≤ 0.05) after applying a one-way ANOVA. The letters “a”, “b”, “c”, “d”, “e”, and “f” next to the values in the table refer to the statistical groupings as a result of the LSD test. When the two values share the same letter, no significant difference is demonstrated; a significant difference is denoted by different letters next to the value.

**Table 4:** Marrubiin content of in vitro obtained and cultivated *M. vulgare* plants and of wild-growing plants.

Sample	Marrubiin, mg/100 g DW
FCP	279.3 ± 17.0 ^a^
FWP	350.0 ± 14.1 ^b^
LCP	120.7 ± 11.0 ^bc^
LWP	138.5 ± 8.8 ^c^
SCP	101.5 ± 14.1 ^d^
SWP	128.5 ± 6.9 ^e^
LSD	13.14

Legend: FCP (flowers of in vitro cultivated plants); FWP (flowers of wild plants); LCP (leaves of in vitro cultivated plants); LWP (leaves of wild plants); SCP (stems of in vitro cultivated plants); SWP (stems of wild plants). Different letters indicate the significance of the analysis, assessed by Fisher’s LSD test (*p* ≤ 0.05) after applying a one-way ANOVA. The letters “a”, “b”, “c”, “d”, and “e” next to the values in the table refer to the statistical groupings as a result of the LSD test. When the two values share the same letter, no significant difference is demonstrated; a significant difference is denoted by different letters next to the value.

**Table 5:** Inhibitory concentrations IC_50_ of *M. vulgare* extracts determined by MTT assay.

Cell Line	LWP	LCP	FWP	FCP	SWP	SCP
HeLa 24 h	>1000	>1000	548.0 ± 16.1 ^a^	692.3 ± 20.1 ^b^	>1000	>1000
HeLa 72 h	235.2 ± 7.2 ^c^	307.0 ± 4.6 ^d^	42.0 ± 1.0 ^a^	168.0 ± 7.0 ^b^	484.4 ± 12.5 ^e^	306.0 ± 7.9 ^d^
HT-29 24 h	>1000	>1000	486.2 ± 11.6 ^a^	757.1 ± 16.7 ^b^	>1000	>1000
HT-29 72 h	377.1 ± 12.1 ^b^	307.5 ± 7.0 ^a^	439.2 ± 8.2 ^c^	330.1 ± 6.1 ^a^	703.3 ± 21.7 ^d^	776.8 ± 19.3 ^e^
MCF-7 24 h	>1000	>1000	732.7 ± 18.0 ^a^	906.8 ± 24.4 ^b^	>1000	>1000
MCF-7 72 h	234.0 ± 7.6 ^a^	375.6 ± 11.7 ^c^	342.8 ± 10.4 ^b^	361.4 ± 10.2 ^c^	>1000	448.9 ± 14.5 ^d^
BALB/3T3 24 h	>1000	>1000	>1000	>1000	>1000	>1000
BALB/3T3 72 h	535.2 ± 17.0 ^a^	669.3 ± 15.5 ^c^	596.0 ± 13.9 ^b^	694.2 ± 17.5 ^c^	>1000	631.4 ± 16.0 ^b,c^

Legend: LWP (leaves of wild plants); LCP (leaves of in vitro cultivated plants); FWP (flowers of wild plants); FCP (flowers of in vitro cultivated plants); SWP (stems of wild plants); SCP (stems of in vitro cultivated plants). Data are presented as mean ± SD. Letters indicate significance of differences between the IC_50_ values of the extracts for each cell line and time point, determined by ANOVA followed by Bonferroni post hoc test. The lowest values are denoted by the letter “a” and subsequent letters are assigned in an ascending order.

## Data Availability

The original contributions presented in this study are included in the article. Further inquiries can be directed to the corresponding authors.
